# The newborn delivery room of tomorrow: emerging and future technologies

**DOI:** 10.1038/s41390-022-01988-y

**Published:** 2022-03-03

**Authors:** Natalie Batey, Caroline Henry, Shalabh Garg, Michael Wagner, Atul Malhotra, Michel Valstar, Thomas Smith, Don Sharkey, the European Society for Paediatric Research (ESPR) Neonatal Resuscitation Section Writing Group, Mara Niemuth, Helmut Küster, Henry Rozycki, Anne Lee Solevåg, Inmaculada Lara-Cantón, Shiraz Badurdeen, Janneke Dekker, Peter Davis, Calum Roberts, Arjan te Pas, Máximo Vento, Burkhard Simma, Marieke den Boer, Heidi Meredith Herrick, Mario Rüdiger, Maxi Kaufmann, Heidi Aichner, Samir Gupta, Willem deBoode, Charles Christoph Roehr, Britt Nakstad, Stuart Hooper, Natalie Batey, Caroline Henry, Shalabh Garg, Michael Wagner, Atul Malhotra, Michel Valstar, Thomas Smith, Don Sharkey

**Affiliations:** 1 Nottingham Neonatal Service, Nottingham University Hospitals NHS Trust, Nottingham, UK; 2 Centre for Perinatal Research, School of Medicine, University of Nottingham, Nottingham, UK; 3 Department of Neonatal Medicine, James Cook University Hospital, Middlesbrough, UK; 4 Division of Neonatology, Pediatric Intensive Care and Neuropediatrics, Department of Pediatrics, Comprehensive Center for Pediatrics, Medical University of Vienna, Vienna, Austria; 5 Monash Newborn, Monash Children’s Hospital and Department of Paediatrics, Monash University, Melbourne, Australia; 6 School of Computer Science, University of Nottingham, Nottingham, UK; 7 Department for Neonatology and Pediatric Intensive Care, Clinic for Pediatric and Adolescence Medicine, Faculty of Medicine, Technische Universität Dresden, Dresden, Germany; 8 Clinic for Pediatric Cardiology, Intensive Care and Neonatology, University Medical Center Göttingen, Göttingen, Germany; 9 Division of Neonatal Medicine, Children’s Hospital of Richmond, Virginia Commonwealth University, Richmond, VA USA; 10 The Department of Paediatric and Adolescent Medicine, Oslo University Hospital, Oslo, Norway; 11 Neonatal Intensive Care Unit, The Department of Paediatric and Adolescent Medicine, Oslo University Hospital, Oslo, Norway; 12 Neonatal Research Group, Health Research Institute and University and Polytechnic Hospital La Fe, Valencia, Spain; 13 Newborn Research Center and Neonatal Services, The Royal Women’s Hospital, Melbourne, VIC Australia; 14 Division of Neonatology, Department of Paediatrics, Leiden University Medical Centre, Leiden, The Netherlands; 15 Department of Paediatrics, Monash University, Clayton, VIC Australia; 16 Department of Paediatrics, Academic Teaching Hospital, Landeskrankenhaus Feldkirch, Feldkirch, Austria; 17 Division of Neonatology, Leiden University Medical Center, Leiden, The Netherlands; 18 Department of Medical Ethics and Health Law, Leiden University Medical Center, Leiden, The Netherlands; 19 Department of Pediatrics, Division of Neonatology, Children’s Hospital of Philadelphia, Philadelphia, PA USA; 20 Saxony Center for Feto-Neonatal Health, Faculty of Medicine, Technische Universität Dresden, Dresden, Germany; 21 Department of Paediatrics, Division of Neonatology and Paediatric Intensive Care Medicine, Medical Faculty, Technical University Dresden, Dresden, Germany; 22 Saxony Center for Feto-Neonatal Health, Medical Faculty, Technical University Dresden, Dresden, Germany; 23 Department of Engineering, Durham University, Durham, UK; 24 Honorary Consultant Neonatologist, University Hospital of North Tees, Stockton-on-Tees, UK; 25 Division of Neonatology, Department of Pediatrics, Sidra Medicine, Doha, Qatar; 26 Kinderarts-neonatoloog, Radboudumc Amalia Children’s Hospital, 6500 HB Nijmegen (804), The Netherlands; 27 National Perinatal Epidemiology Unit Clinical Trials Unit, University of Oxford, Oxford, UK; 28 Newborn Services, Southmead Hospital, Bristol, UK; 29 Department of Pediatrics and Adolescent Health, University of Botswana, Gaborone, Botswana; 30 University of Oslo, Faculty Medicine, Oslo, Norway; 31 Hudson Institute for Medical Research, Department of Obstetrics and Gynaecology, Monash University, Melbourne, VIC Australia; 32 Nottingham University Hospitals NHS Trust, Nottingham Neonatal Service, Nottingham, UK; 33 Centre for Perinatal Research, School of Medicine, University of Nottingham, Nottingham, UK; 34 Department of Neonatal Medicine, James Cook University Hospital, Middlesbrough, UK; 35 Division of Neonatology, Pediatric Intensive Care and Neuropediatrics, Department of Pediatrics, Comprehensive Center for Pediatrics, Medical University of Vienna, Vienna, Austria; 36 School of Computer Science, University of Nottingham, Nottingham, UK

## Abstract

**Abstract:**

Advances in neonatal care have resulted in improved outcomes for high-risk newborns with technologies playing a significant part although many were developed for the neonatal intensive care unit. The care provided in the delivery room (DR) during the first few minutes of life can impact short- and long-term neonatal outcomes. Increasingly, technologies have a critical role to play in the DR particularly with monitoring and information provision. However, the DR is a unique environment and has major challenges around the period of foetal to neonatal transition that need to be overcome when developing new technologies. This review focuses on current DR technologies as well as those just emerging and further over the horizon. We identify what key opinion leaders in DR care think of current technologies, what the important DR measures are to them, and which technologies might be useful in the future. We link these with key technologies including respiratory function monitors, electoral impedance tomography, videolaryngoscopy, augmented reality, video recording, eye tracking, artificial intelligence, and contactless monitoring. Encouraging funders and industry to address the unique technological challenges of newborn care in the DR will allow the continued improvement of outcomes of high-risk infants from the moment of birth.

**Impact:**

- Technological advances for newborn delivery room care require consideration of the unique environment, the variable patient characteristics, and disease states, as well as human factor challenges.
- Neonatology as a speciality has embraced technology, allowing its rapid progression and improved outcomes for infants, although innovation in the delivery room often lags behind that in the intensive care unit.
- Investing in new and emerging technologies can support healthcare providers when optimising care and could improve training, safety, and neonatal outcomes.

## Introduction

Delivery room (DR) management of the newborn focuses on ensuring the physiological process of oxygen delivery transitions from the placenta to the lungs. Most infants make this transition independently, aerating the lungs and establishing a functional residual capacity to allow for gas exchange.^1^ At birth, 85% of term babies breathe within 10–30 s and require no support, 10% respond to stimulation or airway opening, and approximately 5% of infants will require positive pressure ventilation (PPV), 0.3% chest compressions, and 0.05% adrenaline.^2^

Advances in DR technology have enhanced management at birth, facilitating care and reducing factors known to affect neonatal outcomes. Neonatal care has embraced technology, leading to the rapid evolution of the speciality. Despite significant advances in monitoring on intensive care units in recent years, much of this technology has not yet translated into everyday DR care. Adoption barriers include the portability of equipment, evidence of benefit, and the training required for utilisation in high-pressure environments. Furthermore, the DR is a unique setting occurring nowhere else in clinical medicine with factors challenging technology such as fluid-filled lungs, wet skin, 10-fold patient weight differences ranging from <500 g to >5 kg, and a transitioning cardiorespiratory system. It is important to consider the care requirements for the mother and her baby when in the same room as both may need intensive treatment. This requires close communication within teams and with the family. Where possible, technologies should facilitate, rather than impede, family integration and early bonding.

We focus this review on high-income countries where many technologies were designed for, recognising that the greatest burden of morbidity and mortality exists in less well-resourced settings where initiatives such as NEST360^3^ are driving forward technological innovations to improve care. This review discusses the evolution of newborn technologies in the DR with current recommendations, emerging, and potential future technologies.

### Brief history of DR technologies

Paediatric resuscitation has been referenced throughout historical literature with a description of mouth to mouth as far back as 850 BC.^4^ Recognition of establishing respiration during newborn resuscitation, along with the physiological understanding, occurred in the mid-twentieth century. Prior to this, various methods such as intragastric oxygen, respiratory stimulants, hyperbaric oxygen, and rapid hypothermia were all trialled.^5^

Following advances in obstetrics, changes in birth location, and the first neonatal units,^6^ the technology to support newborn care evolved rapidly in the twentieth century. Neonatal physiology research in the 1950s and 1960s^7,8^ improved understanding of acute hypoxia during birth and the management required to alter the pathway of the physiological processes.^6^

Widespread support for positive pressure respiration immediately after birth occurred from the 1950s,^9^ although the method of delivery (face mask vs intubation) remained contentious for some time.^10^ The first newborn resuscitaire by Vickers Medical in 1965 was introduced into practice.^6^ Transcutaneous oxygen saturation monitoring, developed in the 1970s,^11^ was not in mainstream DR use until the past 10 years, especially for preterm infants.^12^

Over the past 20 years, technology use in the DR has evolved and impacted the management of newborns. Recommendations for the early use of suction followed by intubation and the use of rebreathing bags with 100% oxygen for resuscitation has transitioned to the use of mask ventilation commencing in the air for term infants and the titration of oxygen for preterm infants.^13^ Some technologies such as end-tidal CO_2_ detectors have remained part of current practice,^14^ whereas others such as meconium aspirators are less advocated.^2^ Guidelines have mostly been driven by monitoring strategies to support management, this has included the introduction of electrocardiography (ECG) and pulse oximetry monitoring to provide objective measurement in place of more error-prone subjective assessments such as colour or heart rate assessment with a stethoscope.^13,15^

### Current international recommendations

The International Liaison Committee on Resuscitation (ILCOR) publishes a consensus on newborn resuscitation science every 5 years. Current DR design and management strategies are based on these guidelines, this section highlights some of these practice points with a focus on technologies. Key changes to the ILCOR newborn resuscitation guidelines over the past 20 years when technologies started to be integrated into the guidance are outlined in Table 1.

**Table 1:** Key technologies detailed through the last five iterations of the ILCOR newborn resuscitation guidelines.

Year	Key elements	Technologies
2000	• Suction for meconium	• Stethoscope
	• 100% O_2_ for resuscitation	• Exhaled CO_2_
2005	• Less suction for meconium	• T-piece devices
	• Move to air for term resuscitation	• Plastic bags
2010	• Monitor heart rate and oxygen saturations	• Pulse oximeter
	• Consider CPAP	
2015	• Delayed cord clamping	• Pulse oximetry ± ECG
	• Monitor heart rate and oxygen saturations	• Humidified gases
2020	• Reducing invasive ventilation	• ECG for heart rate
	• Focus on monitoring again	• Pulse oximetry for oxygen saturations

As resuscitation guidance has evolved, there has been a focus on reducing neonatal morbidity and mortality, particularly in preterm infants, with aspects of DR care known to improve outcomes. Some of the most important areas related to temperature management, delayed cord clamping (DCC), and the use of monitoring to minimise invasive or harmful treatments, especially those relating to respiratory outcomes.

### Thermoregulation

Temperature management is one of the most important aspects of DR care. ILCOR recommends warming adjuncts (warm blankets, plastic wrapping without drying, cap, thermal mattress) in addition to maintenance of environmental temperature of 23–25 °C for infants <32 weeks gestation, to avoid hypothermia (temperature <36 °C).^15^ This particularly applies to infants <28 weeks gestation since hypothermia is an independent risk factor affecting morbidity and mortality, with increased risk for each degree below 36.5 °C.^16^ The plastic bag/wrap for very preterm infants is perhaps one of the simplest yet cost-effective ways of reducing hypothermia and, when used in combination with other measures such as humidified gas, is associated with lower mortality and severe brain injury.^17^

### Delayed cord clamping

With recent evidence supporting its benefits, DCC is recommended for at least 30 s for infants <34 weeks and 60 s for those ≥34 weeks when immediate resuscitation is not required.^18,19^ Several studies are underway looking at resuscitation with the cord intact. With recommendations for optimal cord management translating into practice,^5^ newer resuscitation platforms allowing stabilisation with the cord intact have been developed and undergone clinical evaluation.^20^

### Monitoring in the DR

Several studies have been conducted looking at the effective provision of resuscitative measures (bag and mask ventilation, provision of adequate tidal volume and inspiratory pressure, cardiac compression) as well as monitoring of various parameters to assess the response to resuscitation (chest wall rise, heart rate monitoring, leak assessment, oxygen delivery, and maintenance of saturation targets). Currently, ILCOR recommends ECG for heart rate monitoring^21^ and pulse oximetry for the management of oxygen titration.^22^ As our understanding of transition at birth increases, it is likely more of these technologies, adapted from the neonatal unit, will evolve further in the DR management of high-risk newborns.

### Survey of opinions on DR technologies

As part of this review, we wanted to understand how clinicians and researchers view the current and future role of technologies in the DR care of high-risk infants. We therefore conducted an online survey via two main channels: (1) direct invitation to the European Society for Paediatric Research Neonatal Resuscitation Section members, and (2) via the neonatal theme of the National Institute for Health Research (NIHR) Children and Young People MedTech Cooperative.^23^ Details of the survey and who completed it can be found in the supplement. Of the 60 respondents, 82% were from Europe and doctors at a senior level. When asked how advances in DR technologies in the past 5–10 years compared to other areas of clinical medicine, only 37% felt it had increased proportionately or significantly. Similarly, only 9% of respondents felt funding for newborn DR technologies had been good or excellent in their region, 65% said it was poorly funded.

The lack of advancement in DR technologies is perhaps a reflection of the level of funding invested in this niche market with relatively small patient numbers compared to other clinical specialties. Evidence for this lack of investment was demonstrated in paediatric high-risk (class III) devices undergoing FDA approvals between 2008 and 2011.^24^ Of the 24 approved for use in children, only 3 (16%) had been studied on patients <18 years of age and so approvals for these high-risk devices were mostly based on adult trials only. This is a pattern previously recognised within paediatric drug development and has resulted in the development of paediatric medical device consortia or cooperatives such as those established in the US^25^ and UK.^26^ This has also resulted in calls to national funders and policymakers to actively encourage paediatric medical device innovation.^27^ The use of pulse oximetry to measure heart rate in the DR is a good example of how we cannot simply adopt devices developed for adults and apply them in the unique environment of the DR on patient populations not previously validated with clinical studies.^28^ Although absolute numbers of babies requiring advanced DR care are small, the need for intervention is not always predictable and therefore a risk at any birth.

**Fig. 1:**
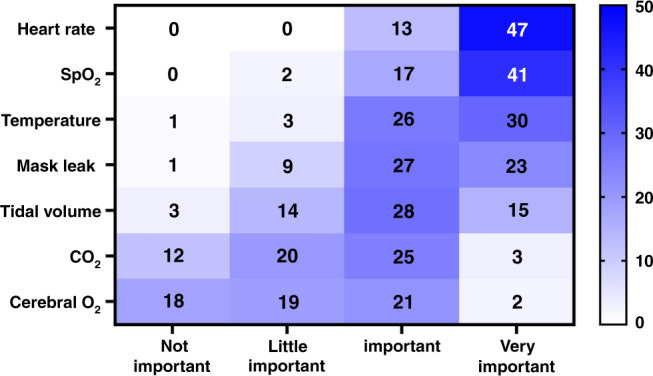
Survey respondents’ (*n* = 60) ranking of the importance of key monitoring measures within the delivery room. SpO_2_ oxygen saturations, CO_2_ carbon dioxide, O_2_ cerebral oxygenation.

There is a multitude of measurements that can be obtained in the DR using technologies. Some, such as temperature, are important when improving outcomes^17^ while the utility of others, such as cerebral oxygenation, are being explored in on-going DR studies.^29^ Our survey found heart rate, oxygen saturations, and temperature to be the most important (Fig. 1). Near infrared spectroscopy (NIRS), for measuring cerebral oxygenation, was ranked the least important reflecting the evolving data on its utility in neonatal care. Respiratory function monitors (RFMs), discussed in more detail later, provide information on ventilation technique and overall was ranked as important by participants.

### Emerging technologies

Although not currently part of newborn resuscitation recommendations, there are a number of exciting emerging technologies that could translate into clinical practice in the DR.

### Respiratory function monitoring

Healthcare professionals (HCPs) provide PPV via a mask using a T-piece or self-inflating bag during newborn airway support.^5,12^ These devices can generate unknown and variable tidal volumes (*V*_T_) depending on mask seal,^30,31^ airway obstruction,^32^ and positioning. A recent study showed *V*_T_ > 6 ml/kg was provided to extremely preterm infants 47.7% of the time.^33^ Uncontrolled, large *V*_T_ are associated with activation of inflammatory cascades,^34,35^ predisposition to bronchopulmonary dysplasia, and higher rates of severe intraventricular haemorrhage (IVH).^36^

Given the difficulty of assessing chest wall expansion, especially in preterm infants,^30,37^ and heart rate estimation errors,^38^ an objective live feedback system of *V*_T_ could potentially improve outcomes.^39,40^ RFMs, such as the Florian and Monivent systems, can provide feedback on tidal volumes, pressure waveforms, and gas flow.^41–43^ RFMs can be used to identify when pressure should be titrated with changes in lung compliance,^42^ if there is suboptimal mask seal^44^ or obstruction,^40^ although device performance can vary.^43^ RFMs improve HCP performance during simulation^39,44–46^ but can also result in incorrect management with inexperienced users.^47^

Schmölzer et al undertook a small clinical trial with RFMs visible or masked and found a positive impact on ventilation quality when the RFM was visible.^40^ Conversely, Sarrato et al. found no difference in short-term surfactant use or intubation need when using RFM.^48^ The MONITOR randomised control trial, comparing blind versus unblinded RFMs in extremely preterm infants at birth, found no differences in the main outcome measures including ventilation breaths within the *V*_T_ range of 4–8 ml/kg.^49^ There were fewer IVHs in the unblinded group although this could be a chance finding and requires additional exploration. These conflicting results raise a number of questions about the usefulness of RFMs and support further evaluation in appropriate clinical trials to understand their usefulness.

### Electrical impedance tomography (EIT)

Developed in the 1980s,^50^ EIT is a functional imaging modality that is safe, non-invasive, and not irradiating. It has been used primarily as a research tool and can determine real-time lung aeration during spontaneous or assisted breaths. Electrodes are placed around the chest allowing alternating electrical currents to be channelled between pairs of electrodes and the remaining electrodes measure conductivity.^51,52^ Tomographic algorithms construct frames, up to rates of 50 Hz, of the lungs as air within the lungs has different conductivity to tissue.

EIT has been used to assess lung changes at birth and in other settings including: well infants,^53^ post surfactant,^54,55^ optimal ventilation strategies,^56^ sustained lung inflations,^57,58^ assessment for extubation,^59^ body positioning,^60^ endotracheal tube placement,^61,62^ effect of suctioning^63^ and pneumothorax identification.^64,65^ EIT allows real-time observation of over-distension and inhomogeneous ventilation,^51^ potentially allowing personalised ventilation strategies.^66^ As with many DR technologies, motion artefact and practicality may limit the usefulness of EIT outside of research settings. Studies using it at birth for preterm infants are also awaited.

### Videolaryngoscopy

Securing the newborn airway in an emergency can often be a stressful procedure^67^ but is an essential neonatal skill. Videolaryngoscopy can be a useful aid that facilitates a higher success rate of the first intubation attempt and shortening hypoxic periods.^68,69^ Videolaryngoscopy is possible with infants of all weights, including birth weights around 500 g.^67^

O’Shea et al. demonstrated the main reasons for failed intubation attempts are oesophageal intubation and the failure to recognise anatomical landmarks, something videolaryngoscopy could address.^70^ Videolaryngoscopy could support teaching and training, improve patient safety and minimise unsuccessful intubation attempts. The most important issues of teaching neonatal intubation are visibility, with learners unable to identify the anatomic structures, and the small airway making it is almost impossible for direct supervision. Videolaryngoscopy overcomes these, improving views and communication between trainee and supervisor.^71,72^ Application of videolaryngoscopy in the DR setting does require robust evaluation to establish if it also translates to fewer intubation attempts, and so better outcomes, and its utility in the management of a difficult airway. As with many technologies, the design of the device is crucial as videolaryngoscopy blades differ from conventional blades^73^ and require a different intubation technique.

### Augmented reality (AR)

AR is a technology that displays holograms in the current physical environment. This technology can enhance training experiences as well potentially reduce errors, improving quality of care and patient outcomes, especially in acute settings.^74^ AR might be helpful for training and clinical situations in the DR, as applied in other settings.^75–77^ Dias et al developed and studied the use of AR-assisted videolaryngoscopy for neonatal intubation. They found, in neonatal nurses with minimal intubation experience, that both AR and standard videolaryngoscopy more than doubled intubation success rates, avoided oesophageal intubations, and significantly reduced time to intubate compared with conventional laryngoscopy.^78^ AR could also be used for remote skill training or even real-time support, where a remote specialist can see the first-person view of the provider wearing the AR headset and provides visual instructions remotely.^79^

### Clinical decision support

Clinical decision support tools utilise computer-controlled software, allowing access to a broad clinical knowledge database. This can be integrated with patient-specific information and suggest recommendations or assessments specific to the patient, supporting complex decision-making. During simulated newborn resuscitations, a decision support tool that provided visual and auditory prompts reduced algorithm deviations including those related to cardiopulmonary resuscitation.^80^ Simple support tools can also be incorporated into tablet or smartphone apps, for example, to improve the accuracy of heart rate assessment.^81^

### Video recording

Recording DR resuscitations with video cameras has been performed for a number of years in some centres.^82–85^ Parents find reviewing resuscitations of their babies as acceptable and valuable.^85^ Recording events can help support training, research, and audit;^86^ this could be particularly helpful during complex resuscitations where the accuracy of documentation is often inadequate^87^ and deviations from algorithms are frequent.^88,89^ Retrospective use of video recording in the DR has been demonstrated to be useful. However, the live viewing of video and its utility in supporting care is one area that requires more research to understand its potential with remote and clinical decision support or the guidance of artificial intelligence (AI) linking other measures with team actions.

### Telemedicine/remote consulting

Telehealth facilitates access to specialised expertise, education and collaboration, and can also reduce transfers and costs. In the DR, telemedicine has the potential to support high-risk deliveries in non-tertiary centres where in utero transfer is not possible. Retrospective studies have found a higher quality of high-risk newborn resuscitations in community hospitals when using telemedicine^90^ and can reduce inter-hospital transfers.^91^ A number of telemedicine neonatal resuscitation simulation studies have been undertaken focussing on team dynamics although not all have shown benefits.^92,93^ Telemedicine in the DR offers an exciting opportunity to enhance training and improve the care of high-risk infants born in non-tertiary centres. Combining telemedicine with AR or eye-tracking warrants exploration to identify additional benefits.

### Eye-tracking

**Fig. 2:**
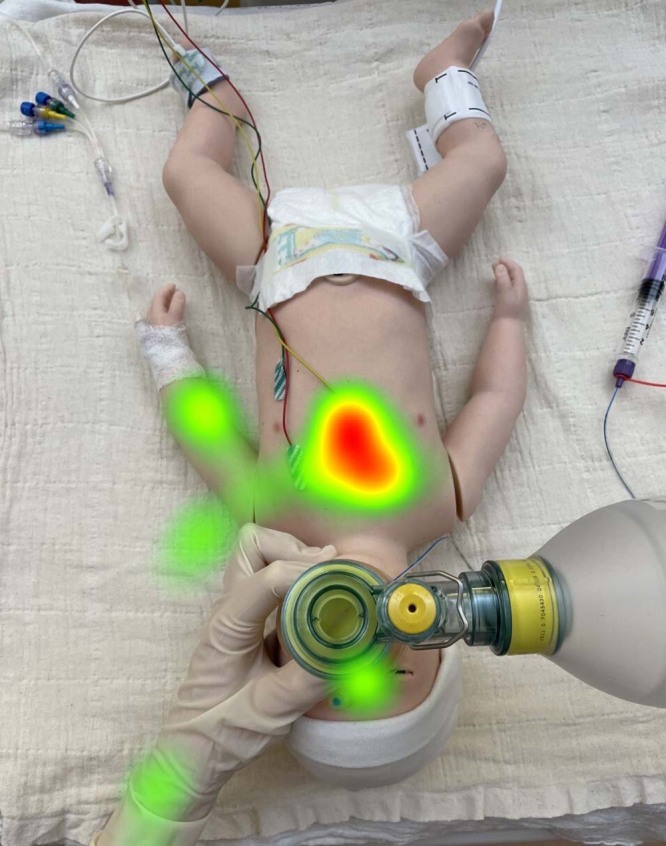
Example heat map after analysis of providers’ gaze behaviour in a simulated resuscitation scenario. Visual attention patterns can be analysed allowing different or new technologies to be evaluated.

The use of eye-tracking glasses enables the analysis of a HCPs visual attention, providing valuable insights into their situational awareness and decision making.^94^ Studies have shown HCPs focus most of their cumulative visual attention time on the infant, followed by monitors, clinical staff, and other physical objects.^95,96^ However, visual attention patterns are different when other technologies or interventions are included (Fig. 2).^97^ Eye tracking studies^98,99^ during simulated and real resuscitations have shown HCPs spend more time looking at the RFM (29 vs 1%), less time looking at the infant (29 vs 46%). Eye-tracking analysis in the DR enables researchers and educators to identify areas of improvement, cognitive (over)load, and the impact of new technologies or monitors. It is also possible to link eye-tracking data with physiological parameters such as heart rate and saturations, facilitating a thorough understanding of HCPs actions and reactions.

### AI in the DR

Algorithms for newborn resuscitation are based on international guidelines and allow a structured approach to stabilisation in the first few minutes of life. Retrospective studies after the introduction of standardised resuscitation programmes have found improvements in survival and neurological impairment.^100^ However, video analysis of real-life resuscitations highlights that teams are rarely able to undertake the desired assessments or interventions in-line with the algorithm timeline.^88,89^ Using real-life video recordings of resuscitations for training can improve the quality of neonatal resuscitation.^88^

The analysis of video recordings of neonatal resuscitation for research, audit, and medical records can be resource heavy in terms of the time required for event categorisation and documentation. Computer vision and AI approaches could significantly speed this process up and even offer real-time feedback of resuscitation progress to support clinical decision-making and avoid delays with treatments. This could then open up the exciting potential to link these data with long-term outcomes creating a digital database for more efficient, big data analyses.

**Fig. 3:**
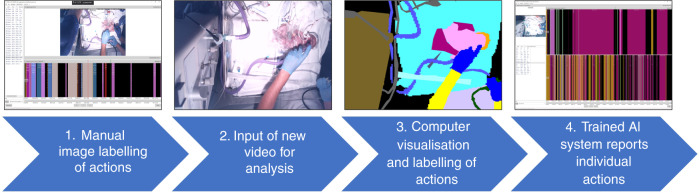
Process of training a computer system to recognise resuscitation actions using computer vision and artificial intelligence (AI). (1) Image training data set manually labelled, categorising actions for the algorithm. (2) New data set (video) is inputted into the AI algorithm for analysis. (3) The algorithm pixelates items to label and track them. (4) Output allows quantification of important actions, their timing, duration, and frequency. Running in real time has the potential to support stabilisation/resuscitation algorithms to avoid deviations or errors especially in settings lacking experienced support.

To explore the potential of AI to analyse complex newborn resuscitation data, Smith et al. used deep-learned superpixels to predict semantic segmentation of videos.^101^ Superpixels are clusters of image pixels that are grouped together by their similarity and this approach allows training of neural networks on a small data set, Smith utilises 23 segmentation classes from just 50 video frames. They trained recurrent neural networks to find the patterns in the video data that align with newborn resuscitation actions (see Fig. 3). The process is initially time consuming as the algorithm needs to be told what each image represents, in terms of action, before learning about the superpixel patterns associated with it. The complexity of newborn resuscitation with 20–30 different actions can be hard to train a system and then for it to accurately predict what is going on. As we learn more about the power of AI in neonatal practice, the opportunity to utilise it in the DR is now a near reality and something that could one day offer real-time support and advice in the care we deliver. For example, AI could be linked with videolaryngoscopy to support real-time intubation when more experienced support was not immediately available.

### Contactless monitoring

A number of groups have looked at contactless monitoring including using video^102^ and radar^103^ in the neonatal unit with mixed results. There are practical issues to translating this into the DR, with the challenging environment and rapidly transitioning newborn, but this perhaps offers an exciting area for future exploration.

### Role of human factors in new DR technologies

The analogy of the Formula One pitstop is often used during resuscitation. This team approach is critical in the DR of the high-risk newborn and requires a full appreciation for the role of human factors in achieving the most successful outcome. Understanding human factors, the interaction between humans and elements within their environment, can help optimise performance and reduce errors.^104^ In the DR, human factors can be optimised^99^ by decreasing cognitive and technical workload and establishing decision support tools. Teamwork during resuscitation has key success factors, including coordination of tasks, a synergistic working process and constructive conflict management at debriefs.^105^ Approaching new technology design and adoption in the DR requires a detailed human factors approach, to avoid cognitive overload and algorithm deviations or errors, although they are rarely considered.^106^ An important example by Pickup et al resulted in the identification of previously unrecognised user needs when developing a newborn heart rate monitor.^107^ Innovators within the DR space need to incorporate human factors research in their developmental pathway to ensure the device performs its function without introducing new, unforeseen risks and distractions.

### Future technologies

We have made great improvements with outcomes of high-risk newborns, in part because of our understanding of transition and DR stabilisation. Newer practices of DCC and resuscitation/stabilisation with an intact placental circulation introduce new challenges for technologies. The interface between the obstetric and neonatal team is a new setting for technologies and requires careful design and application to avoid harm to the mother, for example through infection during caesarean section, and the infant by avoiding hypothermia while still be able to monitor and stabilise. Technologies play a crucial role in providing the attending team with critical information to manage the care of the newborn.

**Fig. 4:**
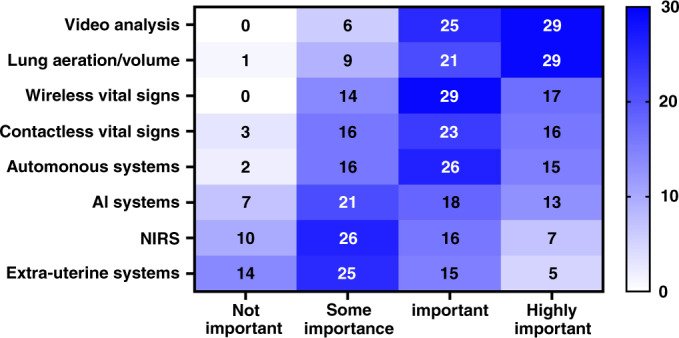
Survey ranking by respondents (*n* = 60) for new technology priorities within the delivery room of the future. AI artificial intelligence, NIRS near infrared spectroscopy.

In our survey, HCPs ranked video analysis and lung aeration as the most important emerging technologies for the next 5–10 years, followed by wireless or contactless vital sign devices (Fig. 4), perhaps reflecting the increasing need to monitor infants during DCC or DR cuddles to avoid trailing wires and the complexity of the stabilisation platform.

To deliver optimal DR care, we need to understand both the clinical status of the newborn and their response to the management we instigate. In the past 20 years, this has become more evident with technology providing us with a wide variety of different measures to guide care. Reducing the complexity of the DR with a whole team circling around a 500 g preterm infant undergoing DCC is going to be important going forward. HCPs ranked wireless monitoring solutions as potentially an important step towards making this happen. There have been some exciting developments with wireless vital signs monitoring in the neonatal unit which offer real potential.^108^ These must be suitably adapted to cope with the different demands of the DR with a transitioning infant undergoing DCC. Wireless monitoring simultaneously measuring key information prioritised by clinicians, i.e. heart rate, oxygen saturations, and temperature, has the potential to reduce sensors, device screens, and subsequent cognitive overload for staff. Wireless monitoring also offers the potential to improve family integrated care with kangaroo care an important component. Both parents and staff see device wires as a barrier to kangaroo care^109^ and this is likely to be the case when facilitating DR cuddles. Contactless monitoring was also highly ranked as a future DR technology.

Newer systems utilising software algorithms, such as autonomous oxygen titration, or AI were felt to be important but perhaps are further downstream as more data become available from studies underway.

Protecting the brain of the foetus and newborn are at the core of obstetric and neonatal care. In neonatology, this goal has resulted in the evolution of the ‘Neuro-NICU’ where monitoring of the brain is a critical component.^110^ NIRS is one of the main modalities currently undergoing intense investigation but even in the Neuro-NICU its role remains unclear but has significant promise. It is therefore not surprising that HCPs did not rank this as an important technology in the DR at this stage. There remain several limitations with neuromonitoring technologies, especially in the DR, with different devices providing different information and uncertainty about translating their use into meaningful outcomes.^111^ Some of these are being addressed, for example targeting cerebral oxygenation in the DR,^29^ but even here we need to consider the impact of overcomplicating team dynamics with multiple sensors, monitors, and subsequent actions, something that is already being studied.^112^

Finally, our survey found extra-uterine systems^113^ were ranked as the least important based on advances suggested, this again may reflect the truly ‘futuristic’ approach to preterm newborn care aiming to mimic the uterine environment and minimise the adverse effects of extreme prematurity. Better monitoring of the breathing and predicting the need for treatments could help reduce some of these adverse effects and technologies may have an important role.^114,115^

## Conclusions

When introducing any new piece of equipment into a time-critical, acutely stressful DR resuscitation scenario, the benefits must be clear. Increasing monitoring modalities in the DR can result in information overload and affect performance. However, emerging technologies show significant promise for safety and the improvement of outcomes following neonatal stabilisation or resuscitation. The addition of technology to the DR will not only impact an individual infant’s care but also enhance the training of HCPs and allow collaborative working across centres. We must now intensify our efforts to deliver new innovative technologies, as other specialties in medicine have done, to improve newborn DR care during the first golden minutes of life, aiming to minimise morbidities that can stay with infants for the rest of their lives.

## Supplementary information

Supplementary Information
