# Supplementary material for: The newborn delivery room of tomorrow: emerging and future technologies
Source: Pediatr Res. 2022 Mar 3;96(3):586–94. doi: 10.1038/s41390-022-01988-y (PMC11499259; doi:10.1038/s41390-022-01988-y)
Supplement: Supplementary file 1 — Supplementary Information [file 41390_2022_1988_MOESM1_ESM.pdf]

Delivery room technology

Showing 60 of 60 responses

Showing **all** responses

Showing **all** questions

Response rate: 60%

1 In which continent are you based?

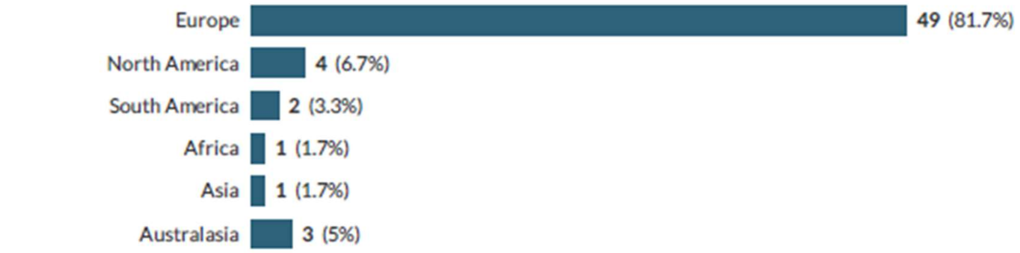

Multi answer: Percentage of respondents who selected each answer option (e.g. 100% would represent that all this question's respondents chose that option)

2 What is your role in newborn care?

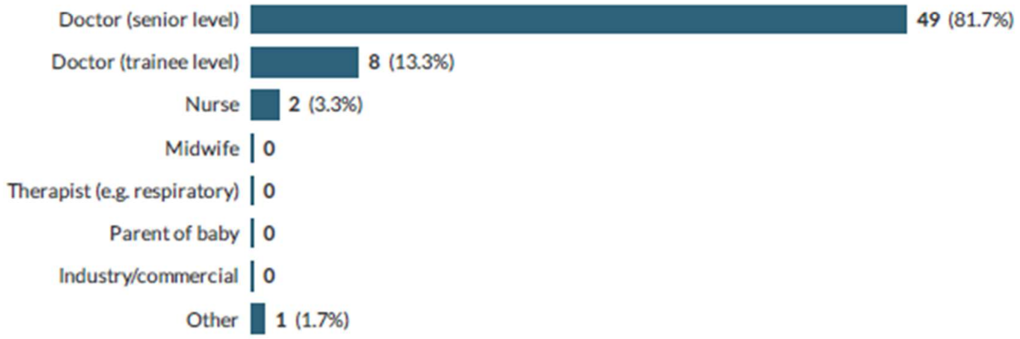

Multi answer: Percentage of respondents who selected each answer option (e.g. 100% would represent that all this question's respondents chose that option)

3 Compared to other areas of clinical medicine, technology in the delivery room in the last 5-10 years has:

3.1 Remained unchanged

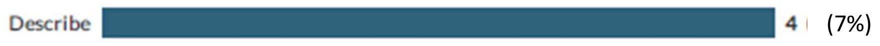

Multi answer: Percentage of respondents who selected each answer option (e.g. 100% would represent that all this question's respondents chose that option)

3.2 Increased a little

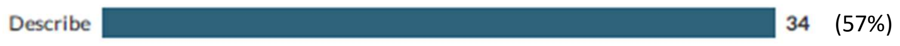

Multi answer: Percentage of respondents who selected each answer option (e.g. 100% would represent that all this question's respondents chose that option)

3.3 Increased proportionately

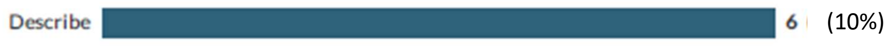

Multi answer: Percentage of respondents who selected each answer option (e.g. 100% would represent that all this question's respondents chose that option)

3.4 Increased significantly

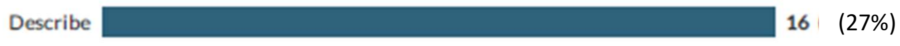

Multi answer: Percentage of respondents who selected each answer option (e.g. 100% would represent that all this question's respondents chose that option)

4 In the last 5-10 years, funding for delivery room technology development and research in my continent has been:

4.1 Poorly funded

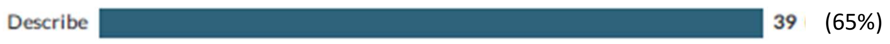

Multi answer: Percentage of respondents who selected each answer option (e.g. 100% would represent that all this question's respondents chose that option)

4.2 Adequately funded

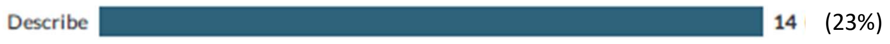

Multi answer: Percentage of respondents who selected each answer option (e.g. 100% would represent that all this question's respondents chose that option)

4.3 Good funding

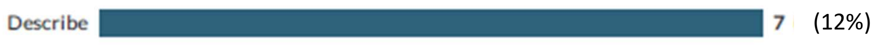

Multi answer: Percentage of respondents who selected each answer option (e.g. 100% would represent that all this question's respondents chose that option)

4.4 Excellent funding

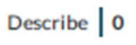

Multi answer: Percentage of respondents who selected each answer option (e.g. 100% would represent that all this question's respondents chose that option)

5 How important are the following measures to you when using technologies in the delivery room?

5.1 Heart rate

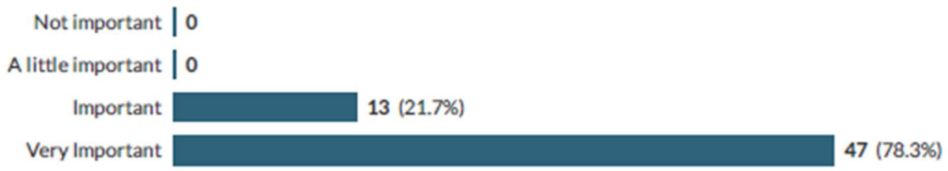

Multi answer: Percentage of respondents who selected each answer option (e.g. 100% would represent that all this question's respondents chose that option)

5.2 Temperature

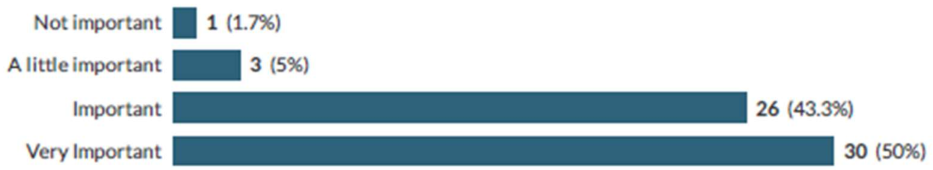

Multi answer: Percentage of respondents who selected each answer option (e.g. 100% would represent that all this question's respondents chose that option)

5.3 Oxygen saturations

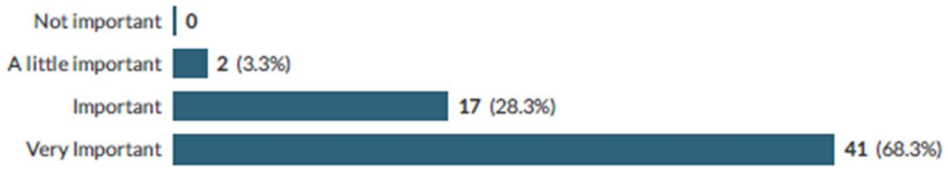

Multi answer: Percentage of respondents who selected each answer option (e.g. 100% would represent that all this question's respondents chose that option)

5.4 Cerebral saturations

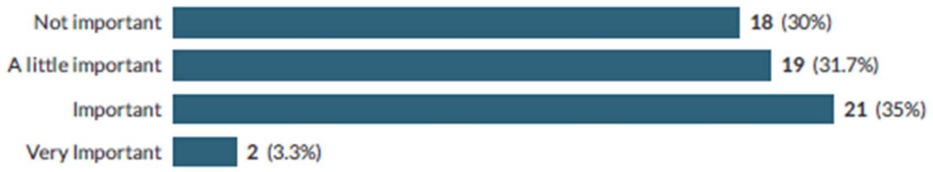

Multi answer: Percentage of respondents who selected each answer option (e.g. 100% would represent that all this question's respondents chose that option)

5.5 Exhaled carbon dioxide

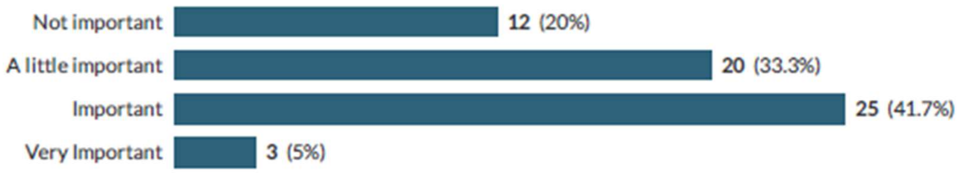

Multi answer: Percentage of respondents who selected each answer option (e.g. 100% would represent that all this question's respondents chose that option)

5.6 Tidal volumes

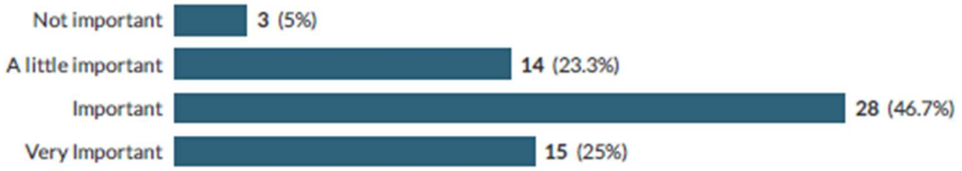

Multi answer: Percentage of respondents who selected each answer option (e.g. 100% would represent that all this question's respondents chose that option)

5.7 Mask leak

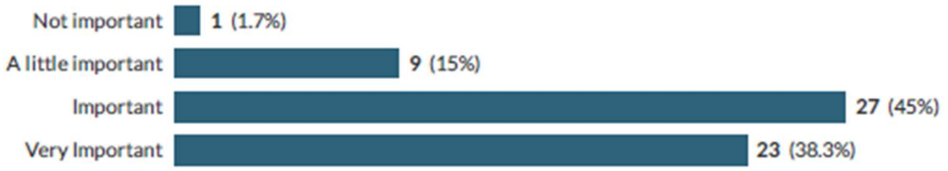

Multi answer: Percentage of respondents who selected each answer option (e.g. 100% would represent that all this question's respondents chose that option)

6 Rate the following delivery room technologies of the future in terms of their importance for the next 5 to 10 years.

6.1 Near-infrared spectroscopy

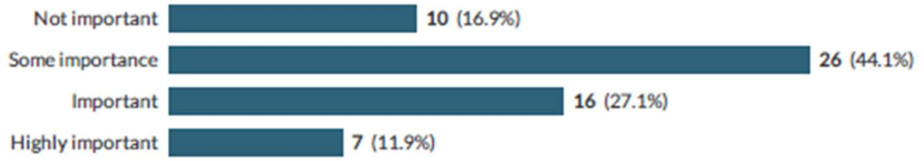

Multi answer: Percentage of respondents who selected each answer option (e.g. 100% would represent that all this question's respondents chose that option)

6.2 Wireless multi-vital signs device

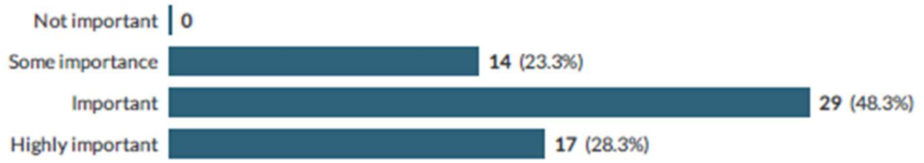

Multi answer: Percentage of respondents who selected each answer option (e.g. 100% would represent that all this question's respondents chose that option)

6.3 Lung aeration/recruitment device

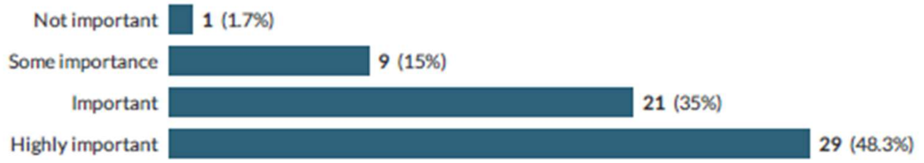

Multi answer: Percentage of respondents who selected each answer option (e.g. 100% would represent that all this question's respondents chose that option)

6.4 Contactless vital signs device

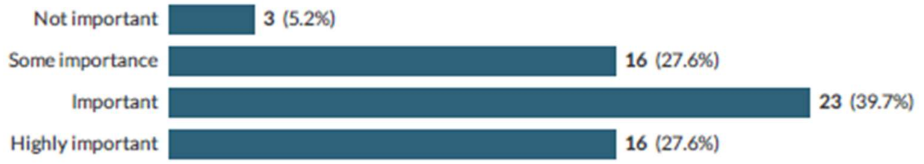

Multi answer: Percentage of respondents who selected each answer option (e.g. 100% would represent that all this question's respondents chose that option)

6.5 Artificial intelligence linking delivery room data with outcomes

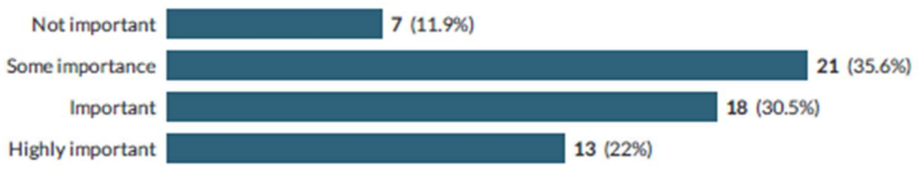

Multi answer: Percentage of respondents who selected each answer option (e.g. 100% would represent that all this question's respondents chose that option)

6.6 Autonomous systems such as inspired oxygen

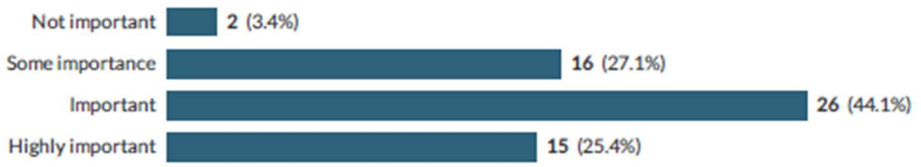

Multi answer: Percentage of respondents who selected each answer option (e.g. 100% would represent that all this question's respondents chose that option)

6.7 Video analysis for training/audit

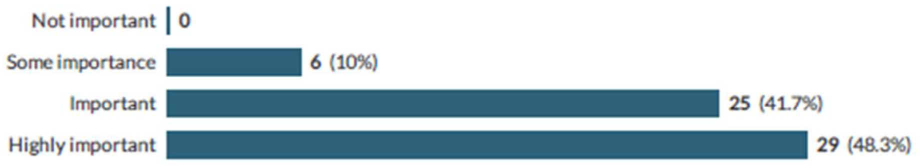

Multi answer: Percentage of respondents who selected each answer option (e.g. 100% would represent that all this question's respondents chose that option)

6.8 Ex-uterine suuport systems (e.g. artificial womb)

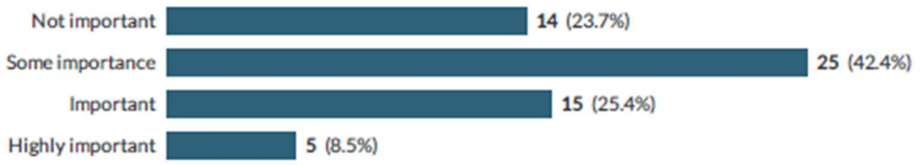

Multi answer: Percentage of respondents who selected each answer option (e.g. 100% would represent that all this question's respondents chose that option)

7 Are there any other technologies you would like to see in the delivery room (state what it would be)?

Showing all 14 responses	
X-ray machinetechnology and Ultrasound technology. Lightsource on the resuscitator cot for fiberoptic bronchoscopy.	765557-765548-80162507
Video Laryngoscopes that are wireless	765557-765548-80169088
non-radiation imaging technologies / ultrasound /	765557-765548-80179298
Telemedicine for delivery room mgmt, ventilation devices (lma?)	765557-765548-80179965
https://jamanetwork.com/journals/jamapediatrics/article-abstract/2780513	765557-765548-80181697
A voice guide or APP to aid in resuscitation.	765557-765548-80195321
Augmented Reality, Decision-support tools, speech recognition, 3D printed devices, stress measurements/biometrics of providers	765557-765548-80196631
Non-invasive cardiac output measurement	765557-765548-80197188
Integrated cardiac function monitoring	765557-765548-80225314
perfusion	765557-765548-80255878
Light assessment to detect pneumothorax, perfusor, Ventilator, incubator/ balance/Apgar	765557-765548-80382399
EIT to monitor lung aeration during transition	765557-765548-80742012
Concord Birth Trolley for physiological cord clamping	765557-765548-80745194
A respiratory function monitor can help in the interpretation of first minutes newborn behaviour and modulate interventions	765557-765548-80947004
